# Differential expression of miRNAs involved in biological processes responsible for inflammation and immune response in lichen sclerosus urethral stricture disease

**DOI:** 10.1371/journal.pone.0261505

**Published:** 2021-12-15

**Authors:** Harjivan Kohli, Brandon Childs, Travis B. Sullivan, Artem Shevtsov, Eric Burks, Thomas Kalantzakos, Kimberly Rieger-Christ, Alex J. Vanni

**Affiliations:** 1 Department of Urology, Lahey Hospital & Medical Center, Burlington, Massachusetts, United States of America; 2 Department of Translational Research, Lahey Hospital & Medical Center, Burlington, Massachusetts, United States of America; 3 Department of Pathology, Boston University School of Medicine, Boston, Massachusetts, United States of America; University of Hyderabad, INDIA

## Abstract

**Purpose:**

To better understand the pathophysiology of lichen sclerosus (LS) urethral stricture disease (USD), we aimed to investigate expression profiles of microRNAs (miRNAs) in tissue samples from men undergoing urethroplasty.

**Methods:**

Urethral stricture tissue was collected from 2005–2020. Histologic features diagnostic of LS were the basis of pathologic evaluation. Foci of areas diagnostic for LS or non-LS strictures were chosen for RNA evaluation. In an initial screening analysis, 13 LS urethral strictures and 13 non-LS strictures were profiled via miRNA RT-qPCR arrays for 752 unique miRNA. A validation analysis of 23 additional samples (9 LS and 14 non-LS) was performed for 15 miRNAs. Statistical analyses were performed using SPSS v25. Gene Ontology (GO) analysis was performed using DIANA-mirPath v. 3.0.

**Results:**

In the screening analysis 143 miRNAs were detected for all samples. 27 were differentially expressed between the groups (false discovery p-value <0.01). 15 of these miRNAs individually demonstrated an area under the curve (AUC)>0.90 for distinguishing between between LS and non-LS strictures. 11-fold upregulation of MiR-155-5p specifically was found in LS vs. non-LS strictures (p<0.001, AUC = 1.0). In the validation analysis, 13 of the 15 miRNAs tested were confirmed to have differential expression (false discovery p-value <0.10).

**Conclusions:**

To our knowledge this is the first study evaluating miRNA expression profiles in LS and non-LS USD. We identified several miRNAs that are differentially expressed in USD caused by LS vs other etiologies, which could potentially serve as biomarkers of LS USD. The top eight differentially expressed miRNAs have been linked to immune response processes as well as involvement in wound healing, primarily angiogenesis and fibrosis.

## Introduction

Lichen Sclerosus (LS) is a chronic inflammatory condition which is presumed to be the underlying etiology of approximately 13–14% of cases of male urethral stricture disease (USD) [1,2]. LS USD is characterized by scarring of the urethra which varies in length and location, occurring anywhere from the urethral meatus to the membranous urethra. The pathophysiology of USD and specifically LS USD is poorly understood. While overall USD urethroplasty success rates range from 84–92% [3,4], successful urethroplasty in patients with LS USD is significantly worse, with recurrence rates ranging from 12–71% [5–9]. Thus, an understanding of the underlying basis of USD is needed to better guide management of LS USD and improve treatment options and outcomes.

Data specific to the pathophysiology of LS USD is limited. A prior study has examined protein expression in LS and non-LS USD and found significantly higher levels of inflammatory markers in strictures caused by LS including CD8 T cells and CCL-4 [10]. Additionally, a recent study evaluated protein expression profiles as a predictor for recurrence in LS USD after urethroplasty and found men with recurrent strictures demonstrating lower levels of inflammation [11]. Although these studies are important in helping define the inflammatory profile of LS USD, the underlying pathophysiology leading to the development of LS USD is still not understood. MicroRNAs (miRNAs) are non-coding genetic material involved in the regulation of gene expression which have shown potential to differentiate behaviors of cancer as well as be involved in regulation of fibrosis. In this study, we sought to examine the pathophysiology of LS and non-LS USD by comparing miRNA expression profiles in men undergoing urethroplasty.

## Methods

### Samples

In this retrospective study, tissue samples were obtained from non-consecutive surgical specimens from 2005 to 2017 from patients undergoing urethroplasty for USD at a single institution under an IRB approved study. Approval was obtained from the IRB of Lahey Hospital and Medical Center. The procedures used in this study adhere to the tenets of the Declaration of Helsinki. All data was analyzed anonymously. Samples were chosen based on adequacy of tissue from paraffin blocks. Pathologists specializing in urologic diseases (EJB & AS) reviewed all samples to create two study groups, LS USD and non-LS USD. Each case was evaluated for 5 typical histologic features of LS: (1) hyperkeratosis, (2) thinning or thickening of the squamous epithelium, (3) attenuation or vacuolar degeneration of the basal cell layer, (4) subepithelial hyalinization and (5) lichenoid lymphoplasmacytic infiltrate. Cases with 3 or more features were regarded as diagnostic of LS, cases with only 2 features were deemed suggestive of LS, while cases with 1 or no features were interpreted as negative for LS [12,13]. For the purpose of this study, two groups were formed based on the following LS score: non-LS strictures (LS score 0–1) and LS strictures (LS score 4–5).

### Study design

The primary analysis of this study was to identify miRNA characteristic of LS USD. To accomplish this an initial screening analysis was performed, using a qRT-PCR array of 752 miRNAs with RNA from 26 formalin-fixed paraffin-embedded (FFPE) urethral stricture specimens. (13 specimens were characterized as non-LS strictures and 13 as LS strictures). A subset of the miRNAs that were significantly differentially expressed in the screening analysis was examined in a subsequent validation analysis, via qRT-PCR, with 23 additional specimens.

### Tissue preparation

After slide review and case classification, 2 mm punch cores were taken from the corresponding paraffin block representing the most diagnostic areas and transferred to a new paraffin block. This was repeated for up to four punches per sample across multiple blocks to create a single individual tissue microarrays (TMA) for each patient. This step was performed to enrich for epithelium since most of the tissue removed during urethroplasty represents soft tissue/erectile tissue rather than epithelium. During the subsequent RNA isolation step, pre and post thin sections (5 μm) were stained with H&E to ensure the diagnostic tissue was sampled on the middle thick section (20 μm) taken for RNA isolation.

### RNA isolation

Total RNA was isolated using the Allprep FFPE DNA/RNA Kit (Qiagen, Hilden, Germany) according to the manufacturer’s instructions using four sequential 20μm FFPE sections per sample. RNA quantity and purity were determined using the Epoch spectrophotometer (BioTek, Winooski, VT, USA) by measuring the OD260 and OD280.

### qRT-PCR

Reactions were performed using miRCURY LNA™ reagents following the manufacturers’ protocols (Qiagen). For each sample, 16ng of total RNA was used in 20μl universal reverse transcription reactions; subsequently 40pg of cDNA was used in each 10μl RT-PCR reaction. Screening analysis was performed using Human miRNome panels I&II version 5 (Qiagen), analyzing 752 miRNAs. Subsequently, a selection of the differentially expressed miRNAs were validated using miRCURY LNA assays prepared in a custom array layout with each miRNA tested in triplicate.

### Data analysis

For the screening analysis miRNA expression levels were normalized to the global mean of each sample for all miRNAs with a Cq <35 for all samples. Expression levels were quantified as x = 2^-ΔCt^ and log2 transformed. Two-sided Welch’s t-tests were performed on the transformed values. Hierarchical clustering using Euclidean distance was performed using GENE-E (Broad) with mean centered values. The expression levels in the validation analysis were normalized to the average of the insignificant control miRNAs: miRs-23a-3p and -107 selected from the screening array dataset using NormFinder [14]. Statistical analyses were performed using SPSS v25 (IBM Corp., Armonk, NY, USA). Significant differences in miRNA expression levels were determined after adjusting for multiple comparisons using the Benjamini and Hochberg False Discovery Rate (FDR) method. Diagnostic accuracy was evaluated by the area under the curve (AUC) for receiver operating characteristic curves.

Gene Ontology (GO) analysis was performed using DIANA-mirPath v. 3.0 [15]. Analyses were performed using the differentially expressed miRNAs with FDR<0.001 and a fold change of <-2 or >2. Predicted gene targets with significant enrichment for these miRNAs, based on the experimentally validated miRNAs interactions derived from TarBase v. 7.0 (University of Thessaly, Volos, Greece), were used. Heat map depicts significant Biological Process clusters resulting from categories union analysis using FDR correction and conservative statistics with a modified Fisher’s Exact Test, p<0.0001.

## Results

### Demographics

In the screening analysis tissue samples from 26 patients total were used which included 13 LS urethral strictures and 13 non-LS urethral strictures. For the validation analysis tissue samples from 23 additional patients were used which included 9 LS urethral strictures and 14 non-LS urethral strictures. There were no significant differences regarding patient age, smoking history, or medical comorbidities such as hypertension, diabetes, and hyperlipidemia between the LS vs non-LS groups; BMI, etiology, and location of stricture were significantly different between the groups (Table 1).

**Table 1:** Patient demographics for all of the samples included in this study.

Characteristic	N = 49	Non-LS (n = 27)	LS (n = 22)	p-value
Age (years)				
median	55	56	53	0.607
range	29–82	29–82	31–73	
Comorbidities				
BMI				
median	30.3	27.3	32.2	0.004
range	21–47.6	21–39.1	22.3–47.6	
Smoking History (%)	19 (38.8)	8 (29.6)	11 (50)	0.238
Diabetes Mellitus (%)	11 (22.4)	6 (22.2)	5 (22.7)	1.0
Hypertension (%)	25 (51)	13 (48.2)	12 (54.5)	0.776
Hyperlipidemia (%)	20 (40.8)	12 (44.4)	8 (36.4)	0.771
Stricture location				<0.001
bulbar (%)	23 (46.9)	22 (81.5)	1 (4.5)	
panurethral (%)	11 (22.4)	1 (3.7)	10 (45.5)	
penile (%)	15 (30.6)	4 (14.8)	11 (50)	
Etiology				<0.001
lichen sclerosus (%)	22 (44.9)	0 (0)	22 (100)	
iatrogenic (%)	7 (14.3)	7 (25.9)	0 (0)	
trauma (%)	8 (16.3)	8 (29.6)	0 (0)	
unknown (%)	12 (24.5)	12 (44.4)	0 (0)	

### Pathology

**Fig 1:**
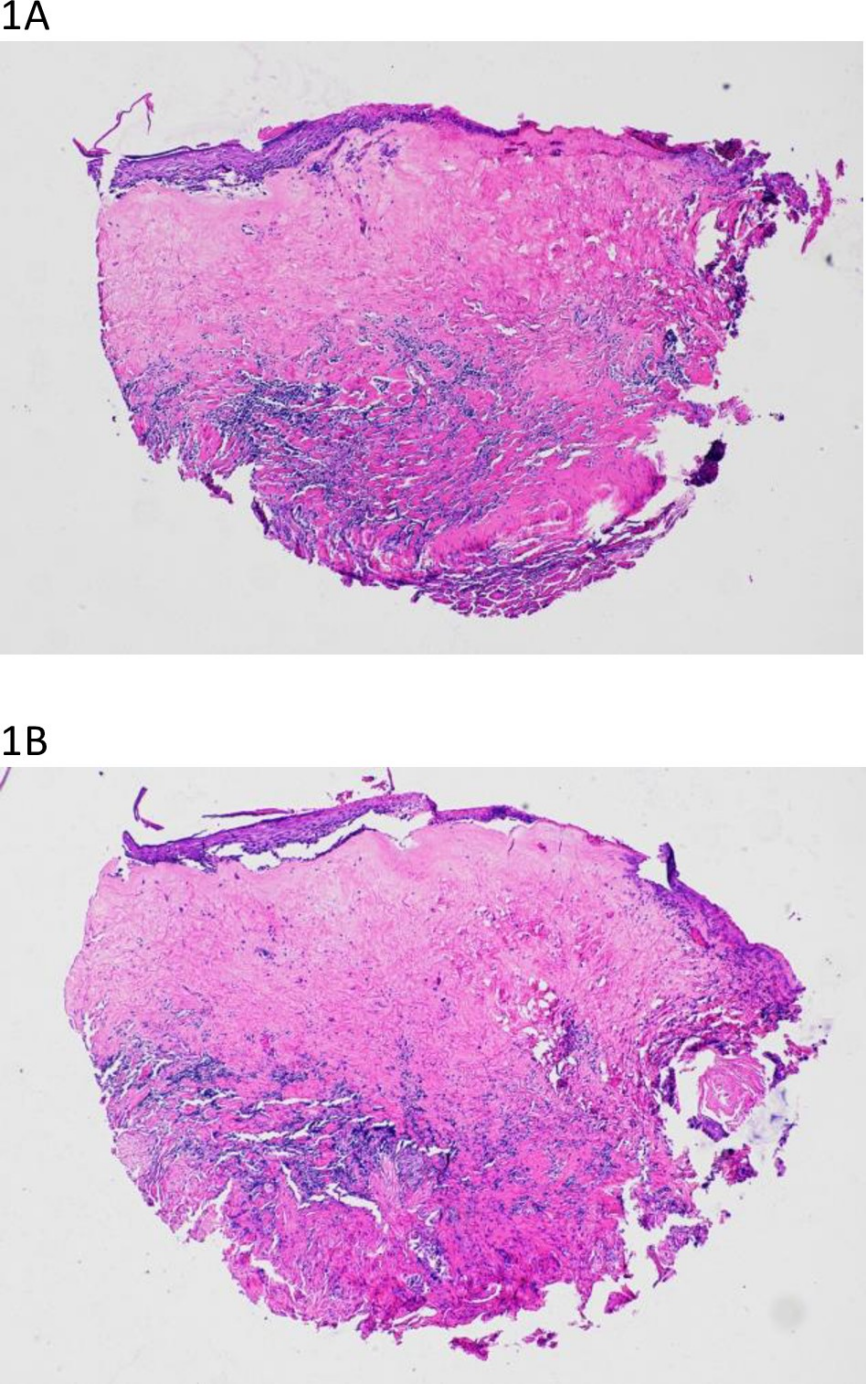
TMA cores. Sectioned before (A) and after (B) RNA isolation showing persistent histologic features typical of LS to include dense subepithelial hyalinization with lichenoid lymphoplasmacytic infiltrate, thinning of the squamous epithelium with focal hyperkeratosis and attenuation of the basal layer.

The pathologic review for each LS case included in the screening study received a score of 5 and the non-LS cases had scores ranging from 0–1: nine patients with a score of 0 and four patients with a score of 1. For the validation analysis the LS cases had scores ranging from 4–5: three patients with a score of 4 and six patients with a score of 5. The non-LS cases had scores ranging from 0–1: seven patients with a score of 0 and seven patients with a score of 1. A subsequent review of the TMA specimens, before and after RNA isolation, revealed consistent scoring with the original case review for all samples (Fig 1).

### Screening analysis: miRNAs expression

**Fig 2:**
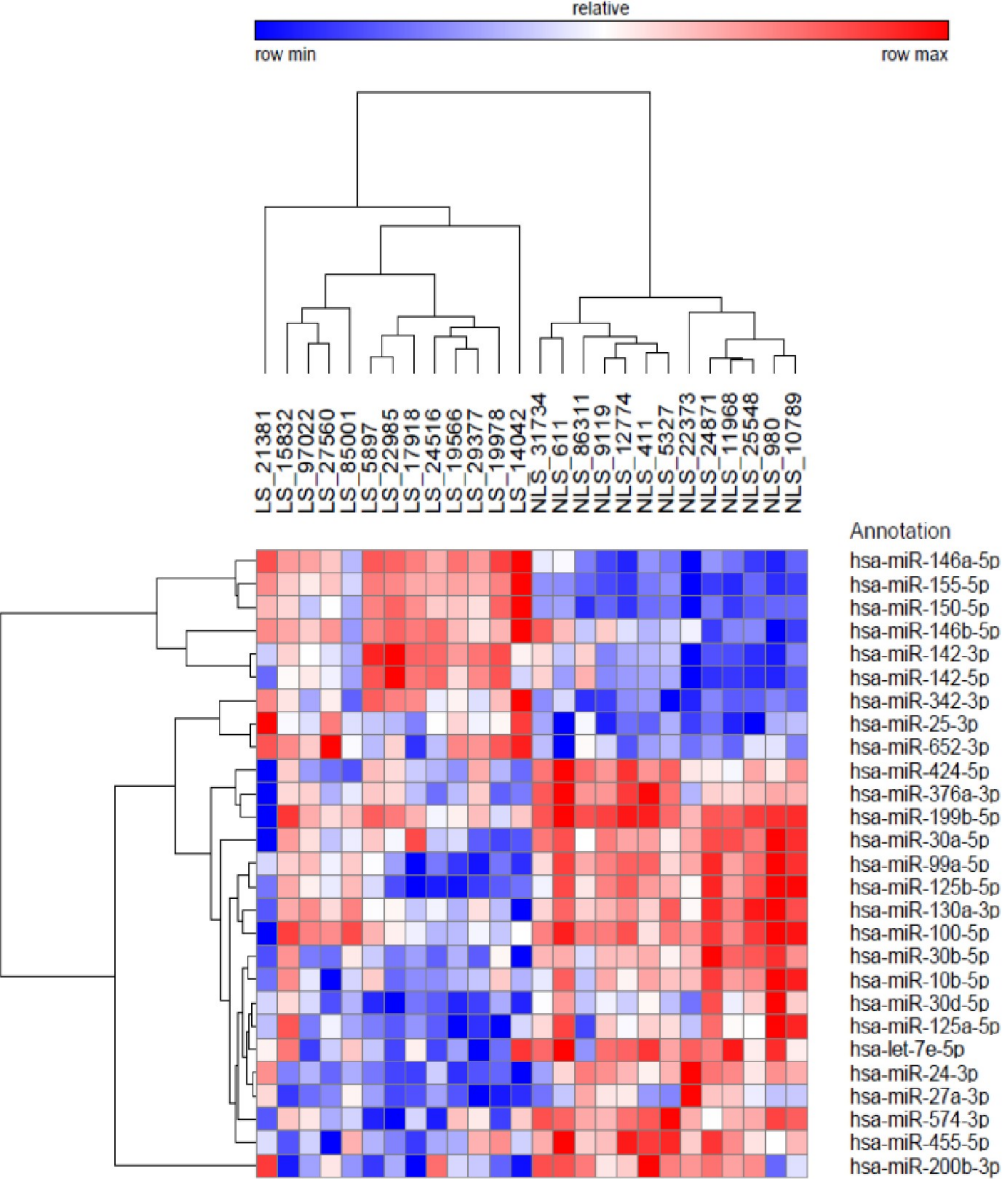
Hierarchical clustering analysis. Hierarchical clustering analysis of the 26 samples included in the screening analysis using the 27 differentially expressed miRNAs (FDR<0.01) identified between LS and non-LS USD.

Of the 752 miRNAs analyzed, a total of 143 miRNAs were detected for all samples (Cq <35). Twenty-seven (27) miRNAs were found to be differentially expressed between the LS and non-LS groups (FDR <0.01; S1 Data). Hierarchical clustering analysis resulted in two distinct clusters differentiating LS and non-LS samples (Fig 2). Of these 27miRNAs, 9 were found to be upregulated in LS, and 18 were found to be downregulated in LS. miR-155-5p specifically was found to be upregulated by 11 fold in LS vs. non-LS strictures (p<0.001, AUC = 1.0). Fifteen of these 27 miRNAs each achieved an area under the curve (AUC)>0.90 for discriminating between LS and non-LS strictures.

### Validation analysis: miRNAs expression

A validation analysis was performed for the 15 significantly differentially expressed miRNAs from the screening analysis with an AUC>0.90 in a second cohort of patients (n = 23). Differential expression was confirmed for 13 of these miRNAs (FDR<0.1, Table 2).

**Table 2:** The median fold change (FC) in miRNAs expression observed in the validation analysis for LS specimens relative to non-LS specimens.

Validation sample set (n = 23)			
Variable	FC	p-value	FDR
miR-574-3p	-1.70	<0.001	0.001
miR-125b-5p	-1.70	0.002	0.015
miR-99a-5p	-1.85	0.003	0.016
miR-376a-3p	-1.53	0.013	0.035
miR-146a-5p	2.46	0.014	0.035
miR-424-5p	-2.06	0.014	0.035
miR-10b-5p	-1.32	0.022	0.048
miR-155-5p	2.03	0.028	0.052
miR-199b-5p	-1.38	0.034	0.052
miR-24-3p	-1.19	0.038	0.052
miR-150-5p	1.75	0.038	0.052
miR-142-3p	1.85	0.045	0.057
miR-30b-5p	-1.17	0.073	0.084
miR-25-3p	1.14	0.479	0.503
miR-342-3p	1.04	0.503	0.503

A negative FC indicates decreased expression of the miRNAs in LS strictures relative to the median expression observed in non-LS strictures. FDR = false discovery rate.

### Gene ontology process prediction

**Fig 3:**
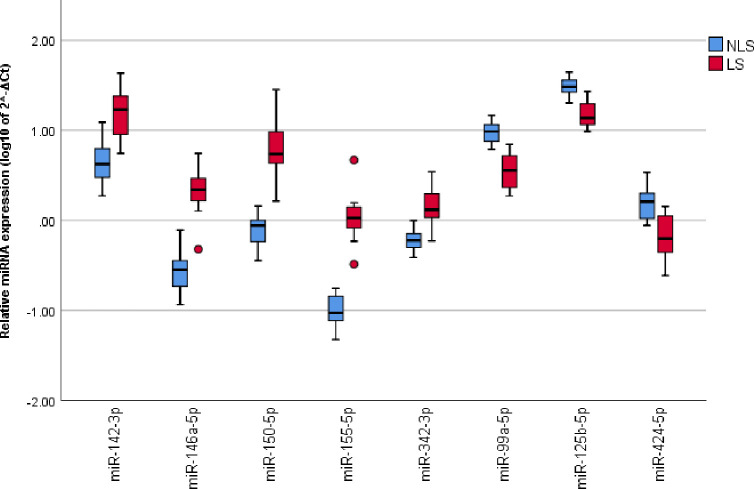
The top eight differentially expressed miRNAs. The top eight differentially expressed miRNAs between the LS and non-LS cohorts from the screening analysis. FDR<0.001 and a FC of <-2 or >2.

**Fig 4:**
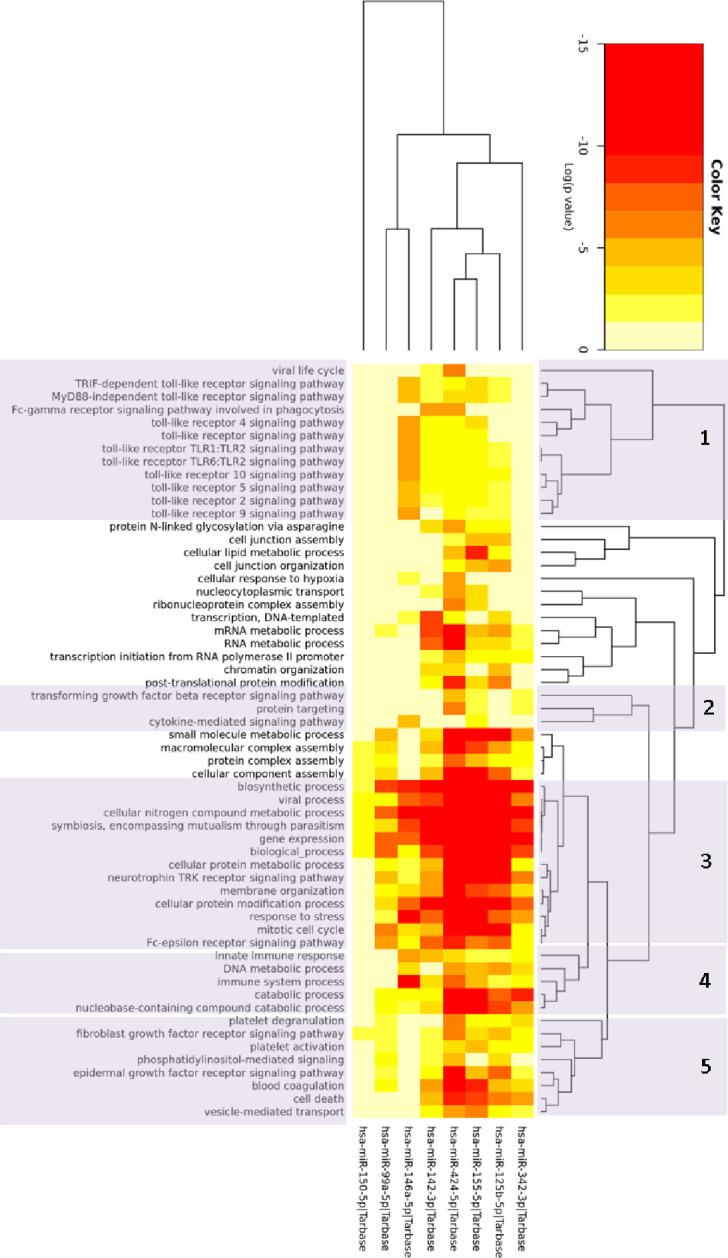
Gene ontology. Two-way hierarchical clustering of the significant gene ontology biologic processes, resulting from the analysis of the top eight differentially expressed miRNAs in the screening analysis. Clusters 1–4 include processes primarily involved in immunity and cluster 5 is comprised of processes related to wound healing. Log of P-values are depicted with greater significance in red.

For the top eight differentially expressed miRNAs in the screening analysis (FDR<0.001, AUC>0.90, and a fold change (FC) of <-2 or >2, Fig 3) 58 significant biologic processes were predicted (Fig 4). The majority of these processes (clusters 1–4) indicate an immune response component differentiating these cohorts; while another grouping (cluster 5) represents processes involved in wound healing, primarily angiogenesis and fibrosis.

## Discussion

To our knowledge this is the first study evaluating USD pathophysiology with miRNA expression profiles in LS and non-LS USD. In our screening analysis we found 27 differentially expressed miRNAs. Of these, 15 miRNAs demonstrated a high probability to differentiate USD caused by LS vs other etiologies (AUC>0.90), and in our validation analysis we confirmed differential expression for 13 of these 15 miRNAs. Several of these miRNAs regulate a number of biologic processes; many have been linked to gene expression in pathways responsible for inflammation, autoimmunity, systemic sclerosis, cell proliferation, apoptosis, and angiogenesis. Additionally, the differentially expressed miRNAs identified in this study could serve as biomarkers of LS; although additional studies are needed to validate their potential. Continued exploration may provide valuable insight for urologists and pathologists in diagnosis, treatment, and possible prevention of LS USD.

Genital LS is a known risk factor for squamous cell carcinoma, and progresses to penile cancer in 2.3–5.8% of male patients [16,17]. Studies of vulvar LS have previously identified some miRNAs of interest. miR-155-5p has been frequently reported to be implicated in the tumorigenesis and progression of multiple types of cancers including colorectal and gastric carcinoma [18,19]. Studies of vulvar LS tissue samples found miR-155-5p to be significantly upregulated; further functional and mechanistic analysis indicated that miR-155-5p promotes fibroblast cell proliferation and inhibits FOXO signaling pathway by negative modulation of both FOXO3 and CDKN1B in vulvar LS [20]. This implicates miR-155-5p as a potential therapeutic target for vulvar LS, and our results indicate a potential role in LS USD as well.

Other miRNAss also found to be upregulated in LS USD compared to non-LS USD have been implicated in the pathophysiology of various types of cancer. miR-146a-5p has been studied in regard to prostate and gastric cancer and is considered to act as a tumor suppressor in these diseases [21–23]. In addition, urinary levels of miR-146a-5p were found to be elevated in bladder cancer patients and were associated with tumor grade and depth of invasion [24]. miR-150-5p, which was also significantly upregulated in LS USD, has been shown to also act as a tumor suppressor in prostate cancer [25]. The roles of miR-146a-5p and mir-150-5p in disease processes more specific to USD, such as cell proliferation and inflammation, are not well established, and require further evaluation to determine their significance in USD.

Investigation of inflammatory mediators in LS USD has been previously performed using evaluation of protein expression related to inflammation [10]. Tissue samples from 81 patients with USD attributable to LS and non-LS causes were evaluated and some inflammatory markers were only found in strictures due to lichen sclerosus. T-cells staining positive for CD8 was significantly higher in strictures due to LS than in those not due to LS, as well as expression of CCL-4. Additionally, tumor necrosis factor-α staining was found to be present only in stricture samples due to LS. Indeed, miR-155-5p, one of the most dysregulated microRNA identified in this study, has been implicated in regulating the expression of these three proteins in other inflammatory conditions [26–28]. Likewise, miR-125b-5p has been shown to modulate the expression of CCL4 [29] and miRs-99a, -142, -146a, and -424 have been associated with TNF α expression [30–33]. Our findings in this study support the theory of inflammatory and possible immune components when the biologic processes of the miRNAs involved are predicted.

Many of the GO processes predicted in this analysis indicate an inflammatory response as a distinguishing feature between these cohorts; in addition, processes involved in fibrosis were predicted. Our findings support some of the defining characteristics of LS: inflammatory dermatosis with subsequent fibrosis complicating the disease [34]. Many of the processes predicted by our findings indicate an innate immune response, primarily involving several of the toll-like receptors. These signaling pathways are some of the most highly characterized within the primary immune system. Specifically, they are essential for the skin’s inflammatory response against invasive pathogens; however, over activation often leads to uncontrolled inflammation and then development of autoimmunity and/or inflammatory skin diseases [35]. In addition, several of the other predicted processes in this study are involved in immune responses (e.g. signaling of Fc receptors and cytokines, viral processes, and parasitism) and provide further insight to the underlying disease mechanisms involved in LS.

This study has notable limitations. Given the paucity of data regarding the pathogenesis of LS, this is meant to be an exploratory study using miRNAs as a surrogate for gene expression; further investigation using gene transcripts is necessary to better elucidate the involved processes. In addition, all samples used in this study represent a highly selected population; not only were samples collected from a tertiary referral center for USD, but in addition all USD tissue samples used were representative of tissue with the highest likelihood of being LS or non-LS, and thus may not represent the true spectrum of inflammatory stricture disease representative of the general LS USD population. Although sample size for this exploratory study is limited, it does not invalidate the profound differences found between the study cohorts.

We hope in subsequent works to evaluate the cellular distribution of these miRNAs by in situ hybridization (ISH) in order to determine the cellular distribution of miRNA expression. LS is a dynamic disease in which early lesions may be difficult to classify and so it is possible that miRNA ISH could be a diagnostic modality used to better classify suggestive lesions that don’t reach definitive histologic criteria to be diagnostic. Moreover, examination of miRNA cellular distribution in these early lesions may also provide valuable insight into the underlying pathogenesis of this poorly understood mucosal inflammatory disease.

Prior studies have demonstrated utility for detection of miRNA expression levels in urine for malignant conditions [24]. Urinary miRNA evaluation could potentially serve as a non-invasive method for evaluation of USD. Development of a urinary test for miRNAs to differentiate LS from non-LS prior to surgery may demonstrate potential to alter the treatment options considered for patients with USD. Additionally, if urinary biomarkers could predict recurrence after urethroplasty, early therapeutic options could be considered to prevent stricture recurrence. Further exploration of the use of urinary biomarkers may have direct applications to the clinical management of patients with both LS and non-LS USD.

## Conclusions

To our knowledge this is the first study to evaluate miRNA expression profiles in LS and non-LS USD, and provides novel insight into the pathophysiology of LS USD. LS urethral strictures demonstrate differential expression of miRNAs involved in processes responsible for inflammation and immune response when compared to non-LS USD. The differentially expressed miRNAs identified in this study suggest excellent predictive value for distinguishing LS vs non-LS USD samples and could potentially serve as biomarkers of LS with further validation in larger cohorts. Further investigation of the gene transcripts involved is necessary to provide better insight into the pathways affected by LS USD.

## Supporting information

S1 Data (DOCX)

## Abbreviations

AUC: Area Under the Curve
FC: Fold Change
FDR: False Discovery Rate
GO: Gene Ontology
LS: Lichen Sclerosus
MiRNA: MicroRNA
TMA: Tissue Microarray
USD: Urethral Stricture Disease
