# Supplementary material for: Differential expression of miRNAs involved in biological processes responsible for inflammation and immune response in lichen sclerosus urethral stricture disease
Source: PLoS One. 2021 Dec 15;16(12):e0261505. doi: 10.1371/journal.pone.0261505 (PMC8673646; doi:10.1371/journal.pone.0261505)
Supplement: S1 Data — (DOCX) [file pone.0261505.s001.docx]

| Supplemental Table | |  |  |  |
| --- | --- | --- | --- | --- |
|  |  |  |  |  |
| Screening analysis results | |  |  |  |
|  | t-test | FDR | AUC | median FC |
| microRNA | p-value | q-value | LS vs NLS | LS vs NLS |
| hsa-miR-155-5p | 0.000 | 0.000 | 1.000 | 11.34 |
| hsa-miR-146a-5p | 0.000 | 0.000 | 0.988 | 7.72 |
| hsa-miR-150-5p | 0.000 | 0.000 | 1.000 | 6.20 |
| hsa-miR-24-3p | 0.000 | 0.000 | 0.941 | -1.40 |
| hsa-miR-99a-5p | 0.000 | 0.000 | 0.964 | -2.71 |
| hsa-miR-125b-5p | 0.000 | 0.000 | 0.947 | -2.22 |
| hsa-miR-30b-5p | 0.000 | 0.000 | 0.935 | -1.58 |
| hsa-miR-342-3p | 0.000 | 0.000 | 0.953 | 2.18 |
| hsa-miR-574-3p | 0.000 | 0.001 | 0.970 | -1.63 |
| hsa-miR-142-3p | 0.000 | 0.001 | 0.917 | 4.00 |
| hsa-miR-424-5p | 0.000 | 0.001 | 0.905 | -2.58 |
| hsa-miR-455-5p | 0.000 | 0.001 | 0.888 | -1.58 |
| hsa-miR-25-3p | 0.000 | 0.002 | 0.935 | 1.37 |
| hsa-miR-376a-3p | 0.000 | 0.002 | 0.923 | -2.41 |
| hsa-miR-10b-5p | 0.000 | 0.002 | 0.899 | -1.67 |
| hsa-miR-142-5p | 0.000 | 0.002 | 0.876 | 2.43 |
| hsa-miR-30a-5p | 0.000 | 0.002 | 0.882 | -2.11 |
| hsa-miR-30d-5p | 0.000 | 0.003 | 0.888 | -1.25 |
| hsa-miR-146b-5p | 0.001 | 0.004 | 0.870 | 2.92 |
| hsa-miR-652-3p | 0.001 | 0.004 | 0.870 | 1.55 |
| hsa-miR-199b-5p | 0.001 | 0.005 | 0.917 | -1.94 |
| hsa-miR-130a-3p | 0.001 | 0.005 | 0.882 | -1.57 |
| hsa-let-7e-5p | 0.001 | 0.005 | 0.864 | -1.41 |
| hsa-miR-125a-5p | 0.001 | 0.007 | 0.876 | -1.31 |
| hsa-miR-200b-3p | 0.001 | 0.008 | 0.828 | -3.68 |
| hsa-miR-27a-3p | 0.002 | 0.009 | 0.858 | -1.30 |
| hsa-miR-100-5p | 0.002 | 0.009 | 0.864 | -1.79 |
|  |  |  |  |  |
| negative fold change (FC) indicates lower expression in LS | | | |  |
